# HSD17B13: A Potential Therapeutic Target for NAFLD

**DOI:** 10.3389/fmolb.2021.824776

**Published:** 2022-01-07

**Authors:** Hai-bo Zhang, Wen Su, Hu Xu, Xiao-yan Zhang, You-fei Guan

**Affiliations:** ^1^ Advanced Institute for Medical Sciences, Dalian Medical University, Dalian, China; ^2^ Department of Pathophysiology, Shenzhen University Health Science Center, Shenzhen, China; ^3^ Health Science Center, East China Normal University, Shanghai, China

**Keywords:** 17β-HSD13, NASH, SNPs, lipid droplet, hepatocarcinoma

## Abstract

Nonalcoholic fatty liver disease (NAFLD), especially in its inflammatory form (steatohepatitis, NASH), is closely related to the pathogenesis of chronic liver disease. Despite substantial advances in the management of NAFLD/NASH in recent years, there are currently no efficacious therapies for its treatment. The biogenesis and expansion of lipid droplets (LDs) are critical pathophysiological processes in the development of NAFLD/NASH. In the past decade, increasing evidence has demonstrated that lipid droplet-associated proteins may represent potential therapeutic targets for the treatment of NAFLD/NASH given the critical role they play in regulating the biogenesis and metabolism of lipid droplets. Recently, HSD17B13, a newly identified liver-enriched, hepatocyte-specific, lipid droplet-associated protein, has been reported to be strongly associated with the development and progression of NAFLD/NASH in both mice and humans. Notably, human genetic studies have repeatedly reported a robust association of HSD17B13 single nucleotide polymorphisms (SNPs) with the occurrence and severity of NAFLD/NASH and other chronic liver diseases (CLDs). Here we briefly overview the discovery, tissue distribution, and subcellular localization of HSD17B13 and highlight its important role in promoting the pathogenesis of NAFLD/NASH in both experimental animal models and patients. We also discuss the potential of HSD17B13 as a promising target for the development of novel therapeutic agents for NAFLD/NASH.

## Introduction

With the development of the economy and society, the morbidity and mortality of metabolic diseases caused by overnutrition and lifestyle changes are still increasing and likely will continue to rise. As a central organ of glucose and lipid metabolism, the liver plays a critical role in developing many metabolic diseases, including nonalcoholic fatty liver disease (NAFLD), type 2 diabetes mellitus (T2DM), dyslipidemia, and obesity. Long-term nutrition overload causes both systemic and hepatic metabolic disturbance and increases the risk of developing chronic liver diseases. NAFLD is the most common chronic liver disease worldwide (Younossi et al., 2015). The typical pathology of NAFLD is characterized by hepatocellular steatosis, lobular inflammation, hepatocyte ballooning, and fibrosis. NAFLD represents a wide spectrum of liver disorders ranging from nonalcoholic fatty liver (NAFL; simple steatosis) to nonalcoholic steatohepatitis (NASH) with inflammation and hepatocyte injury, which further drives fibrosis, eventually leading to cirrhosis and hepatocellular carcinoma (HCC) (Friedman et al., 2018). Among different stages of NAFLD, NASH has been recognized as a critical stage responsible for an increasing proportion of cirrhosis, HCC, and end-stage liver disease (Peng et al., 2020).

NAFLD has a high prevalence in both developing and developed countries. It is estimated that the total number of NASH patients in the United States, Japan, and five European Union countries (Great Britain, France, Germany, Italy, and Spain) will reach 18 million in 2027. Meanwhile, the predicted number of NASH patients in the United States will reach 27 million in 2030 with an increase of 56% (Estes et al., 2018). The prevalence of NAFLD/NASH increased from 23.8 to 32.9% during 1998–2018 in China (Zhou et al., 2020). It is projected that the NASH population in China will further increase to 48.26 million in 2030 (Estes et al., 2018). As a result, from 2004 to 2016, the registration number of both male and female liver transplantation caused by NASH was increased by 80% (Noureddin et al., 2018). In Japan, NASH has become the third leading cause of death in patients with type 2 diabetes mellitus (T2DM) (Nakamura et al., 2017). It has been well-documented that although NAFL carries a very low risk of adverse outcomes, the presence of NASH significantly increases both liver and non-liver-related consequences. In addition to several severe hepatic complications, including cirrhosis, liver failure, and HCC, NASH is considered as a multi-organ disease and is associated with a markedly increased risk of developing cardiovascular disease, type 2 diabetes, hypertension, chronic kidney disease, and extrahepatic malignancy (Friedman et al., 2018). However, despite substantial advances in clarifying the underlying mechanisms of NAFLD and identifying therapeutic targets for this disease in recent years, there are currently no effective therapies available for patients.

Pathologically, NAFLD is defined as an abnormal accumulation of neutral lipids such as triglycerides and cholesterol ester stored in lipid droplets (LD), a subcellular organelle in hepatocytes (Sharma and John, 2021). LDs are complex and metabolically active organelles. Alteration of LD’s biogenesis, growth, or degradation affects their sizes and numbers in liver cells. Excessive biogenesis and constant growth of LDs are the most distinctive characteristics of NAFLD and are closely associated with the progression of NAFLD towards NASH and cirrhosis (Scorletti and Carr, 2021). LDs are composed of a neutral lipid core surrounded by a phospholipid monolayer associated with various proteins. In the past decade, human genome-wide association studies (GWAS) have revealed that a group of genes encoding LD-associated proteins such as FIT2 (fat storage inducing transmembrane protein 2), adipose triglyceride lipase (ATGL; PNPLA2), and PNPLA3 (patatin-like phospholipase domain containing 3) plays an important role in the pathogenesis and progression of NAFLD. Among them, the best-characterized genetic risk factor for NFALD is a missense variant of PNPLA3 (rs738409, C > G, p.I148M). The 148M allele was associated with increased hepatic triglyceride contents and elevated serum ALT levels in a multi-ethnic population-based cohort. The 148M variant evades ubiquitylation and proteasomal degradation and accumulates on lipid droplets where it competes with ATGL to interact with ABHD5, leading to reduced ATGL activity (BasuRay et al., 2019; Gao et al., 2019; Kozlitina, 2020). These findings have provided compelling new insights into the pathogenesis of NAFLD and highlighted a novel strategy for the development of therapeutic agents by directly targeting LD-associated proteins.

By using a comparative proteomic approach, we screened differentially expressed LD-associated proteins between histologically normal and biopsy-proven steatotic human liver samples and first reported hydroxysteroid 17β-dehydrogenase 13 (HSD17B13) as a novel liver-enriched, hepatocyte-specific, LD-associated protein in the liver, where it promotes hepatic lipogenesis and the pathogenesis of NAFLD (Su et al., 2014). Subsequently, multiple human GWAS studies have been performed in different ethnic populations, in which genetic variants of HSD17B13 are reproducibly associated with the full spectrum of NAFLD and influence the risk and fate of NAFLD and severity of steatosis, inflammation, and fibrosis (Anstee et al., 2020). Here, we briefly overview the discovery, tissue distribution, and subcellular localization of HSD17B13 and highlight the important role in promoting the pathogenesis of NAFLD/NASH in both experimental animal models and patients. We also discuss the potential of HSD17B13 as a promising target for developing novel therapeutic agents for NAFLD/NASH.

## Cloning, Tissue Distribution and Subcellular Localization of HSD17B13

**FIGURE 1:**
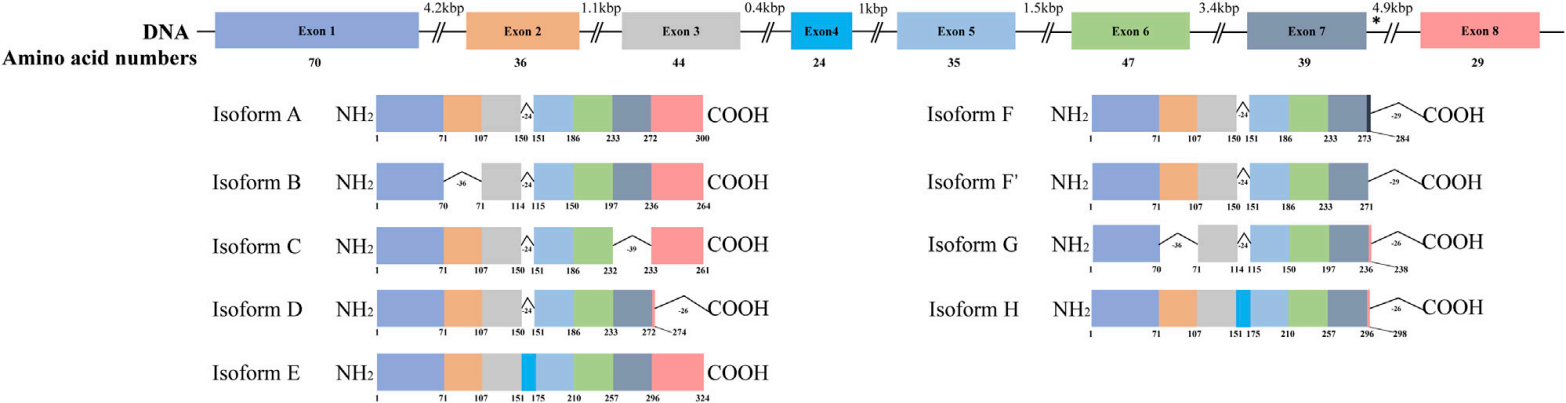
Schematic diagram of human HSD17B13 splice variants. Human hydroxysteroid 17-beta dehydrogenase 13 (HSD17B13) is located at chromosome 4q22.1 with 8 exons and encodes 9 different protein isoforms (HSD17B13 isoform A-H). The exons were boxed and the introns were shown as broken black lines. The newly identified exon 4 was boxed in blue. In isoform F, the C-terminal 52 amino acids (aa233-284) were encoded by the exon 7 and a part of intron 7 sequence (nt12665-13502). The asterisks indicated the position of A insertion from rs72613567 in human HSD17B13 gene. The genetic variant rs72613567 T/T gives rise to a wild type protein, while the variant rs72613567:TA results in a truncated loss-of-function of HSD17B13 protein.

**FIGURE 2:**
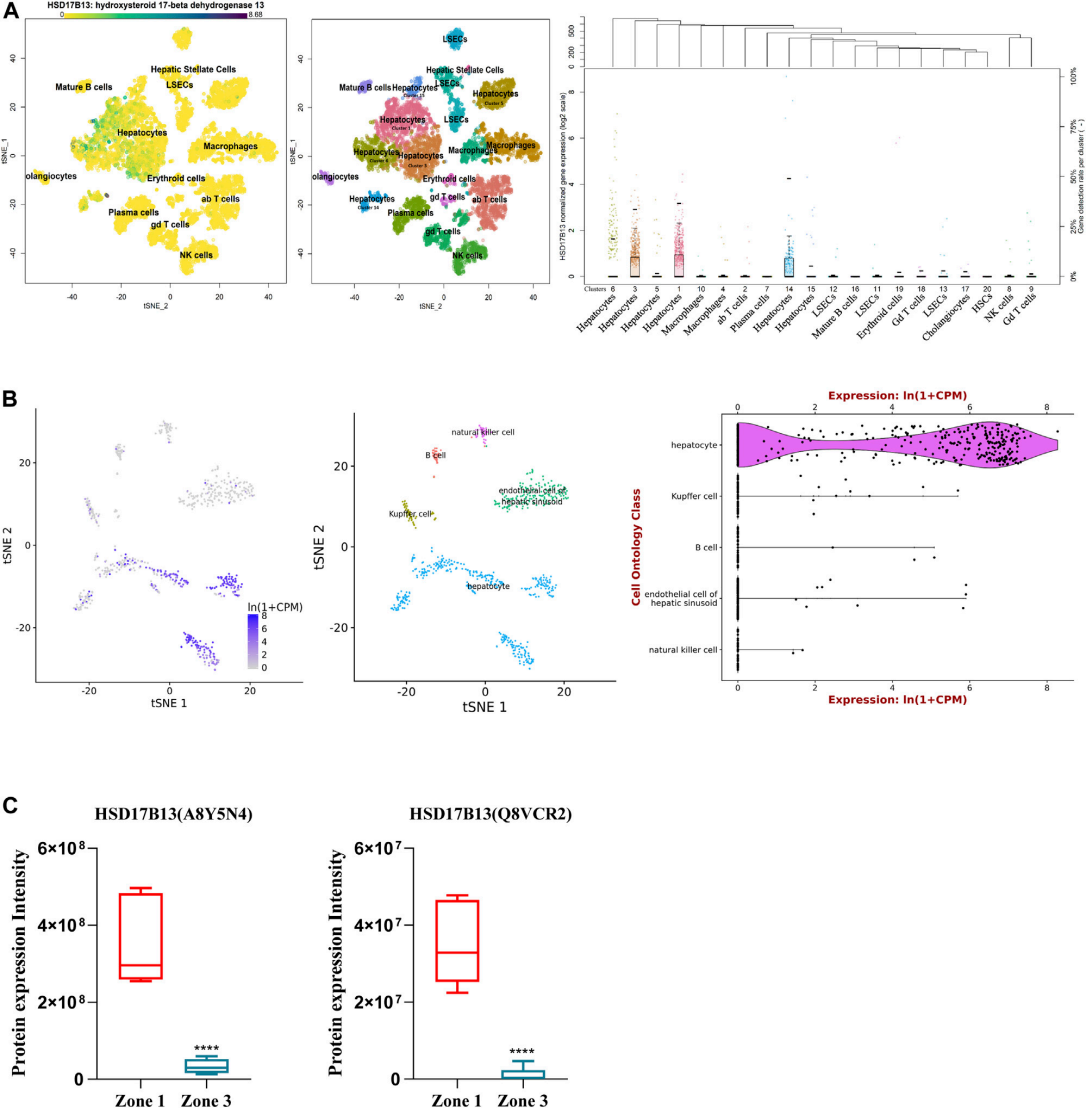
Distribution of human and mouse HSD17B13 protein in liver cells. **(A)** Single cell RNA-sequencing (scRNA-seq) analysis showing that human HSD17B13 is mainly expressed in the hepatocytes. **(B)** Liver cell scRNA-seq analysis demonstrating that mouse HSD17B13 mRNA is highly abundant in the hepatocyte. **(C)** Two protein isoforms of mouse HSD17B13 (A8Y5N4, Q8VCR2) is abundantly expressed in the zone 1 of the liver.

HSD17B13 belongs to the HSD17B family with NAD(P)H/NAD(P)^+^-dependent oxidoreductase activity that catalyzes the interconversion between 17-ketosteroids and 17-hydroxysteroids to maintain the balance between less potent (17-keto) and more potent (17β-hydroxy) forms of estrogens and androgens (Poutanen and Penning, 2019). There are 15 members of this family identified. Most of them are related to the activation or inactivation of sex hormones (HSD17B1, HSD17B2, HSD17B3, HSD17B5, HSD17B6), and the other members are involved in fatty acid metabolism, cholesterol biosynthesis, and bile acid production (Saloniemi et al., 2012). The HSD17B13 gene was firstly cloned from the human liver cDNA library in 2007 and initially named SCDR9 (short-chain dehydrogenase/Reductase 9) (Liu et al., 2007). The human HSD17B13 (SCDR9) gene spans about 17 kb in the human genome and is located at chromosome 4q22.1 with 8 exons and 7 introns. It encodes 9 different protein isoforms through alternative splicing with the functional role of each isoform largely unknown (Figure 1). HSD17B13 proteins possess two conserved motifs seen in all SCDR family members. One is the TGXGXXXG motif related to NAD(P) (H) binding, and the other is the YXXXK motif critical for its catalytic activity. The protein sequence of HSD17B13 shares a high degree of homology (73.7%) with HSD17B11, another member of the HSD17B family. The HSD17B13 gene is 13 kb upstream of the HSD17B11 gene, suggesting these two genes may result from gene duplication (https://www.ncbi.nlm.nih.gov/gene/345275). Interestingly, HSD17B11 is also a lipid droplet-associated protein, although it is more broadly expressed than HSD17B13 (Horiguchi et al., 2008; Yu et al., 2018). The molecular mass of HSD17B13 proteins is between 22 and 33 kDa due to multiple isoforms. It has been reported that human HSD17B13 is most abundantly expressed in the liver, with low levels in the ovary, bone marrow, kidney, brain, lung, skeletal muscle, bladder, and testis (Liu et al., 2007). Single-cell RNA-seq (scRNA-seq) analysis showed that human HSD17B13 is mainly localized in hepatocytes, with very low expression in other liver cells such as cholangiocytes, macrophages, hepatic stellate cells, liver sinusoidal endothelial cells (LSECs), T cells, and plasma cells (Figure 2A) (MacParland et al., 2018). Together, these findings demonstrate that human HSD17B13 is a liver-enriched, hepatocyte-specific, lipid droplet-associated protein.

Similarly, the mouse HSD17B13 gene is also mainly expressed in the liver, with much lower levels in other organs such as the ovary, kidney, brain, lung, and testis (Su et al., 2014). By searching a publically available single-cell RNA sequencing database (https://tabula-muris.ds.czbiohub.org/) which sequenced more than 100,000 cells from 20 mouse organs and tissues (Tabula Muris Consortium et al., 2018), it was found that HSD17B13 was highly expressed in mouse hepatocytes, with very low levels in Kupffer cells, LSECs, B cells and NK cells (Figure 2B). In addition, we analyzed a quantitative shotgun proteomics database of liver cells in different zonation of mouse liver (Berndt et al., 2021), which yielded that both mouse HSD17B13 splice variant proteins, i.e., A8Y5N4 and Q8VCR2, were selectively expressed in periportal hepatocytes at high levels (PPHs, Zone 1), with much less expression in pericentral hepatocytes (PCHs, Zone 3) (Figure 2C). However, it remains unclear whether a similar zonal expression pattern of HSD17B13 exists in the human liver, which requires further investigation.

As a liver-specific protein, HSD17B13 is selectively expressed in hepatocytes, where it is exclusively localized on the surface of lipid droplets. After transfecting an expression vector harboring a human HSD17B13-GFP fusion cDNA in Huh7 cells, it was found that HSD17B13 was exclusively present on the surface of LDs (Su et al., 2014). Consistently, it was also reported that in HepG2 cells, HSD17B13 was co-localized with ADRP, a LD-specific protein (Ma et al., 2019). These findings were further confirmed by immunohistochemical studies showing HSD17B13 was predominantly localized on the envelope of LDs in both human and mouse livers (Su et al., 2014). In addition, Ma Y et al. further characterized the molecular basis for LD targeting of human HSD17B13 and found that the N-terminal sequence AAs 1–28 is required for its LD localization (Ma et al., 2020). To date, little is known about the mechanisms involved in the regulation of HSD17B13 expression. A recent report showed that mouse HSD17B13 was induced by liver X receptor α (LXRα) in a SREBP1-dependent manner, suggesting it may contribute to the metabolic actions of LXRα in the liver (Su et al., 2017). In addition, in terms of its biochemical function, HSD17B13 has been shown to possess both SCDR and retinol dehydrogenase (RDH) activity (Su et al., 2014; Ma et al., 2019). Altogether, these results clearly indicate that HSD17B13 is an evolutionally conserved hepatocyte-specific LD-associated protein with similar tissue distribution and subcellular localization between species.

## HSD17B13 in NAFLD/NASH

As a liver-enriched, hepatocyte-specific, and LD-associated protein, HSD17B13 may play an important role in the regulation of liver lipid droplet biogenesis, growth, and degradation. It is anticipated that aberrant expression and dysfunction of HSD17B13 may contribute to the pathogenesis of chronic liver diseases, especially NAFLD. Two independent studies revealed that the hepatic expression of HSD17B13 was significantly induced in patients with NAFLD relative to that in healthy individuals (Su et al., 2014; Ma et al., 2019). In addition, upregulated HSD17B13 protein was mainly located in the LD subcellular fraction and on the surface of LDs (Su et al., 2014). In line with this finding, in a choline deficient diet (CD)-induced murine NASH/NAFLD model, HSD17B13 expression was found to be significantly increased (Mitsumoto et al., 2017). These findings suggested a close association of increased LD HSD17B13 levels with NAFLD development. In support, the HSD17B13 expression level was also found to be up-regulated in the livers of type 2 diabetic db/db mice and high-fat diet (HFD)-induced obese mice compared with control mice. Moreover, adenovirus-mediated hepatic overexpression of human HSD17B13 for 4 days resulted in accelerated LD biogenesis and excessive neutral lipid accumulation in the mouse liver (Su et al., 2014). In addition, it has also been reported that HSD17B13 may mediate LXRα activation-associated liver steatosis *via* a SREBP1-dependent mechanism (Su et al., 2017). These findings provide convincing evidence that increased hepatic HSD17B13 expression promotes liver LD biogenesis and NAFLD development. Surprisingly, a recent study on HSD17B13 gene knockout mice showed that HSD17B13 deficiency failed to protect the liver from high-fat diet-, Western diet- and alcohol exposure-induced steatotic damages, indicating that HSD17B13 deficiency might not have a protective role in murine NAFLD models (Ma et al., 2021). Similarly, another group reported that mice deficient of the HSD17B13 gene spontaneously developed late-onset fatty liver at the age of 9 months under normal chow (Adam et al., 2018). These unexpected results currently lack mechanistic explanations, which require further investigation.

Although murine models have produced inconsistent results, human GWAS studies have uncovered robust and reproducible associations between HSD17B13 gene variations and the natural history of NAFLD/NASH (Anstee et al., 2020). In a large-scale cohort study of exon-sequencing for 46,544 people of European ancestry, Abul-Husn and colleagues reported a common loss-of-function of HSD17B13 SNP (rs72613567: TA), with an insert of A allele adjacent to the splice site of exon 6 which caused the protein to be truncated prematurely. The presence of this variant reduced the risk of chronic liver diseases including NFALD, ALD and HCC. In human liver samples, the rs72613567:TA variant was associated with a reduced risk of nonalcoholic steatohepatitis, but not steatosis. They also found possible interactions between the HSD17B13-rs72613567:/A allele and PNPLA3-rs738409G-allele, which directly affected the liver PNPLA3 mRNA expression level and reduced PNPLA3 p.I148 activity (Abul-Husn et al., 2018).

The association between HSD17B13 rs72613567:TA and decreased severity of the chronic liver disease has been repeatedly confirmed in several other ethnic populations (Gellert-Kristensen et al., 2019; Ma et al., 2019; Pirola et al., 2019). In addition, Stender’s group has recently reported that an unweighted genetic risk score combining three genetic variants including PNPLA3p.I148M, TM6SF2 p.E167K and HSD17B13 rs72613567 for fatty liver disease conferred up to a 12-fold higher risk of cirrhosis and up to a 29-fold higher risk of HCC in individuals from the general population in Europe (Gellert-Kristensen et al., 2020). Moreover, in patients with biopsy-proven NAFLD, PNPLA3 G/-, TM6SF2 T/- and HSD17B13 TA/- carriage were found to be associated with the severity of NAFLD, and combining PNPLA3 (rs738409, *p* = 0.0076), HSD17B13 (rs72613567), and TM6SF2(rs10401969, *p* = 0.0076), MBOAT7 (rs641738) with clinical data further increased the accuracy for predicting the severity of NASH and/or advanced fibrosis (Ioannou, 2021; Paternostro et al., 2021). Meanwhile, another study explored the impact of the HSD17B13 variant in 1,153 non-Hispanic NASH patients in the United States. The result showed that the protection of HSD17B13 rs72613567 on NASH and fibrosis might be limited to specific individuals who were aged ≥45 years, women who had class ≥2 obesity or diabetes, and those with PNPLA3 rs738409 CC genotype (Vilar-Gomez et al., 2021). These findings demonstrate that the protective effect of HSD17B13 rs72613567:TA could be influenced by the coexistence of other genetic variants such as PNPLA3 and TM6SF2 and the presence of clinical risk factors including diabetes, obesity, and alcohol consumption.

In the past years, two additional loss-of-function human HSD17B13 variants have been identified, including a protein-truncating variant rs143404524 (p.Ala192LeufsTer8) (Kozlitina et al., 2018) and a low-frequency missense variant rs62305723 G > A (p.P260S) (Ma et al., 2019). Both variants confer a partial loss of function of HSD17B13 and are associated with decreased disease severity of NAFLD. Interestingly, a few human HSD17B13 genetic variants located in various non-coding areas, including rs10433879, rs6834314, rs3923441, rs9992651, and rs72613567 were also reported to be associated with reduced severity of NAFLD (Ma et al., 2019; Namjou et al., 2019; Whitfield et al., 2019; Anstee et al., 2020; Satapathy et al., 2021) (Table 1). It is worth mentioning that HSD17B13 rs6834314 (A > G) was a loci initially identified by GWAS in 61,089 individuals. This variant was found to be associated with plasma concentrations of liver enzymes, including ALT, GGT, and ALP (Chambers et al., 2011) and liver fat contents (Ma et al., 2019). Collectively, both preclinical animal studies and human genetic surveys have provided compelling new insights into the pathogenesis of NAFLD/NASH and support the possibility that HSD17B13 may represent as a potential target for the treatment of NAFLD/NASH and chronic liver disease.

**TABLE 1:** A partial list of human HSD17B13 genetic variants associated with NAFLD.

Variant Id	Location	Change	Variant type	Alleles	Transcript change	Amino Acid change	Molecular consequences
rs10433937	4p13:87308948	AAAAGCT [T/A/C/G]ATC	SNV	T > A, T > C, T > G	—		intron variant
rs10433879	4p13:87309988	GAAACCT [G/C]TCTCTA	SNV	G > C	—	—	intron variant
rs72613567	4p13:87310241–87310242	TGTACTT [-/A]CTTCTGT	indel	dupA	—	—	splice donor variant
rs62305723	4p13:87310277	TACGATG [G/A]AACAA	SNV	G > A	c.778C > T	Pro260Ser	missense variant
rs61748262	4p13:87317092	CACTCAC [C/A/T]CAAA	SNV	C > A, C > T	c.450G > T, c.450G > A	Trp150Cys, Trp150Ter	missense variant, nonsense (stop gained)

SNV, Single nucleotide variation; indel, Insertion and Deletion; dupA, duplicate Adenine. Data from NCBI Variation Viewer (http://www.ncbi.nlm.nih.gov/variation/view).

## HSD17B13 SNPs in Other Chronic Liver Diseases

In addition to NAFLD/NASH, the human HSD17B13 rs72613567: TA variant was also reported to be associated with a reduced risk of other chronic liver diseases. A whole-exon sequencing (WES) study involving 46,544 European participants found that the HSD17B13 rs72613567: TA variant was related with a markedly reduced risk of alcoholic liver disease (by 42% among heterozygotes and by 53% among homozygotes) and alcoholic cirrhosis (by 42% among heterozygotes and by 73% among homozygotes) (Abul-Husn et al., 2018). Consistently, the genetic variant rs72613567 of the HSD17B13 gene was also found to reduce alcohol-related liver disease (ALD) risk in the Chinese Han population. In a case-control study including 769 ALD patients and 767 healthy controls, the HSD17B13 rs72613567:TA allele was associated with a decreased risk of ALD by 19% (Chen et al., 2020). Furthermore, a combination of another HSD17B13:rs68343143 variant and two other genetic variants (PNPLA3:rs738409 and TM6SF2:rs10401969) can predict the development of alcohol-related cirrhosis in heavy drinkers (Whitfield et al., 2021). Importantly, the risk of hepatocellular carcinoma was also decreased in patients heterozygous (*OR* = 0.65) and homozygous (*OR* = 0.28) for the HSD17B13 rs72613567:TA allele (Abul-Husn et al., 2018). A genetic score combining three genetic variants (PNPLA3 p.I148M, TM6SF2 p. E167K and HSD17B13 rs72613567) for fatty liver disease conferred up to a 12-fold higher risk of cirrhosis and up to a 29-fold higher risk of HCC in individuals from the general population (Gellert-Kristensen et al., 2020). Altogether, these results indicate that the genetic variants, especially the loss-of-function of SNP of HSD17B13 rs72613567, are very likely related to a reduced risk of ALD, alcohol-related cirrhosis, and hepatocarcinoma.

## Summary and Perspective

NAFLD has become the leading cause of liver cirrhosis and hepatocarcinoma in developed countries. Its prevalence in many developing countries such as China is also rapidly rising, with rates approaching those of Western countries (Wang et al., 2014). The burden of NAFLD is increasing at an alarming rate. NAFLD-related liver inflammation, fibrosis, cirrhosis, and hepatocellular carcinoma represent a major and increasing threat to human health (Adams et al., 2017). For NAFLD, especially in its severe form NASH, there is currently no specific and effective treatment or medicine for patients. The pathogenesis of NAFLD has not been fully understood but is believed to result from a combination of environmental and genetic risk factors. In the past years, GWAS and whole exon sequencing (WES) studies have identified several genetic risk factors closely associated with the development, progression, and severity of NAFLD. PNPLA3 was the first and the best-characterized genetic risk factor, which regulates lipid droplet lipolysis in hepatocytes and is the most strongly associated genetic variant linked to NASH (Romeo et al., 2008; Friedman et al., 2018). Subsequently, a few other genetic variants, including TM6SF2, MBOAT7, and HSD17B13, have been uncovered and also considered as important genetic modifiers of NAFLD (Meroni et al., 2021).

Since HSD17B13 was first reported as a pathogenic factor for NAFLD in 2014 (Su et al., 2014), tremendous progress has been made in our understanding of its contribution to the development and progression of NAFLD. Although there are inconsistent reports in animal models, a few HSD17B13 genetic variants have been reproducibly confirmed in several large clinical and population-based studies with different ethnicities to be linked to the full spectrum of NAFLD (Kozlitina, 2020; Meroni et al., 2021) (Table 1). Currently, the natural substrate(s) and metabolite(s), biological roles, and underlying mechanisms of HSD17B13 in liver lipid metabolism remain incompletely understood. Nevertheless, based on the promising and convincing results from human genetic studies and some of preclinical works, HSD17B13 has been tested in patients as a therapeutic target. Alnylam Pharmaceuticals has recently launched a phase I clinical trial (https://clinicaltrials.gov/ct2/show/NCT04565717), aiming to develop an RNAi-based therapeutic approach to target HSD17B13 for the treatment of NAFLD/NASH. In addition, Arrowhead has also launched a phase I clinical trial (https://clinicaltrials.gov/ct2/show/NCT04202354) in 2019. The result showed that ARO-HSD significantly down-regulated liver HSD17B13 mRNA and protein expression and markedly reduced serum ALT and AST levels. Meanwhile, Inipharm announced that INI-678, a potent and selective small-molecule HSD17B13 inhibitor, showed reduction in key markers of liver fibrosis (α-SMA, COL-I) in a human liver cell-based 3D liver-on-a-chip-model (https://inipharm.com/). These studies are still at the early stage of clinical trials, and their effects on NAFLD/NASH require long-term observation. Moreover, the HSD17B13 genetic variant rs72613567, alone or in combination with other genetic variants and clinical risk factors, may hold great promise to predict individuals who are more susceptible to NAFLD and at high risk for NASH.
